# Erratum for “Prevalence of a Novel Immunogenic Feline Erythrocyte Antigen (FEA 6) and Expression Patterns Between FEAs”

**DOI:** 10.1111/jvim.70192

**Published:** 2025-07-24

Bajon, F., Arsenault, J. and Blais, M.C. (2025), Prevalence of a Novel Immunogenic Feline Erythrocyte Antigen (FEA 6) and Expression Patterns Between FEAs. *J Vet Intern Med*, 39: e70094. https://doi.org/10.1111/jvim.70094.

In the above‐mentioned article, in the Abstract Animals section the word “hundread” appears instead of “hundred.” The correct spelling of the number is “Two hundred seven.”

We apologize for this error.

